# Hypoxic/Ischemic Inflammation, MicroRNAs and δ-Opioid Receptors: Hypoxia/Ischemia-Sensitive Versus-Insensitive Organs

**DOI:** 10.3389/fnagi.2022.847374

**Published:** 2022-05-09

**Authors:** Yimeng Chen, Yichen He, Shuchen Zhao, Xiaozhou He, Dong Xue, Ying Xia

**Affiliations:** ^1^Department of Urology, The Third Affiliated Hospital of Soochow University, Changzhou, China; ^2^Shanghai Key Laboratory of Acupuncture Mechanism and Acupoint Function, Fudan University, Shanghai, China

**Keywords:** MicroRNAs, hypoxic/ischemic inflammation, δ-opioid receptor (DOR), organs’ differential responses, NLRP3 inflammasome

## Abstract

Hypoxia and ischemia cause inflammatory injury and critically participate in the pathogenesis of various diseases in various organs. However, the protective strategies against hypoxic and ischemic insults are very limited in clinical settings up to date. It is of utmost importance to improve our understanding of hypoxic/ischemic (H/I) inflammation and find novel therapies for better prevention/treatment of H/I injury. Recent studies provide strong evidence that the expression of microRNAs (miRNAs), which regulate gene expression and affect H/I inflammation through post-transcriptional mechanisms, are differentially altered in response to H/I stress, while δ-opioid receptors (DOR) play a protective role against H/I insults in different organs, including both H/I-sensitive organs (e.g., brain, kidney, and heart) and H/I-insensitive organs (e.g., liver and muscle). Indeed, many studies have demonstrated the crucial role of the DOR-mediated cyto-protection against H/I injury by several molecular pathways, including NLRP3 inflammasome modulated by miRNAs. In this review, we summarize our recent studies along with those of others worldwide, and compare the effects of DOR on H/I expression of miRNAs in H/I-sensitive and -insensitive organs. The alternation in miRNA expression profiles upon DOR activation and the potential impact on inflammatory injury in different organs under normoxic and hypoxic conditions are discussed at molecular and cellular levels. More in-depth investigations into this field may provide novel clues for new protective strategies against H/I inflammation in different types of organs.

## Introduction

MicroRNAs (miRNAs) are a class of 21∼25-ribonucleotide single-strand non-coding RNA molecules which is endogenously expressed in eukaryote cells (Guo et al., 2014). It has been well recognized that miRNAs play an important role in the post-transcriptional regulation of genes by targeting mRNA molecules. Through base-pairing with the 3′untranslated regions (3′UTRs), miRNAs modulate direct cleavage and/or translational repression of target mRNAs (Bartel, 2009), thus regulating wide spectrum of processes in physiological and pathological conditions. Their regulatory networks are very complex, partially because miRNA expression varies dramatically in different tissues in response to different cell stimulations.

Hypoxic/ischemic injury is a process in which blood flow cessation and oxygen deprivation occur in the body, organs, or cells. Metabolic waste products are accumulated due to the starvation of oxygen and nutrients, leading to profound tissue dysfunction and damage (Zhao et al., 2018). More specifically, hypoxia is a state of low oxygen supply, resulted from a decrease in partial pressure of oxygen, inadequate oxygen transport, or the inability of the tissues/organs to uptake oxygen from the blood. In contrast, ischemia is defined as inadequate blood flow to the tissues, resulting in the deprivation of oxygen and glucose, as well as other substances in the blood (Chao and Xia, 2010; Yang et al., 2015). In this review, the term of “H/I injury” is used to describe the general influences of hypoxic and/or ischemic insult, because hypoxia and ischemia processes often occur one after another. A wide range of pathophysiological processes have been reported to be associated with H/I injury, including ATP depletion, calcium overload, mitochondrial dysfunction, oxidative stress, and etc. (Eltzschig and Eckle, 2011; Chen et al., 2019). More recently, emerging evidence suggests that H/I inflammation, a quick pathological response to oxygen/blood flow depletion, has a major impact on H/I injury through pro-inflammatory cytokines and their signaling pathways (Chen et al., 2020c; Sikora et al., 2021; Troscher et al., 2021).

Because of energy imbalance, H/I insult induces pro-inflammatory events including microglia activation, cytokines production, and immune cell development via various biological processes such as apoptosis and oxidative reaction (Chen et al., 2020c). Different organs may suffer from H/I inflammation in different ways, thus leading to various H/I-related diseases. H/I neuroinflammation induces brain injury in ischemic stroke and post-stroke epilepsy (Li X. et al., 2020; Troscher et al., 2021; Xue Y. et al., 2021), while H/I inflammation leads to other pathophysiological events in peripheral organs, including heart failure, acute kidney injury, and hepatocellular damages (Nangaku and Eckardt, 2007; Klune and Tsung, 2010; Frohlich et al., 2013; Yang et al., 2015). On the other side, some beneficial reaction may go through the regulation of inflammatory events. For example, long term limb remote ischemic conditioning could decrease blood pressure via inflammation regulation (Gao et al., 2021). Studies have demonstrated the vital roles of miRNAs in the regulation of gene expression under H/I condition, not only in H/I-sensitive organs such as kidney, heart, and brain, but also in the H/I-insensitive organs such as liver and muscle (Nallamshetty et al., 2013; Bertero et al., 2017). H/I inflammation is mediated, at least partially, by the miRNA-induced repression of gene expressions. The alternations of miRNA expression in response to H/I injury differ widely in different organs due to their differential sensitivities to the reduction of oxygen and blood flow. Numerous studies have shown the changes of miRNA expression profiles along with the potential targets and inflammatory pathways upon H/I insult in specific organs (Tables 1, 2). However, few studies have compared the common changes and the differences of miRNA profiles between H/I-sensitive and -insensitive organs.

**TABLE 1:** H/I-induced changes of miRNAs in the kidney with defined target genes.

H/I regulated miRNAs	Species	Target genes	Functions	References
**Up-regulated by H/I**				
miR-21	Mouse	CBS, CSE	Promote macrophage M1 inflammatory phenotype	Pushpakumar et al., 2021
miR-23a	Mouse	A20	Activate macrophages and promote tubulointerstitial inflammation	Li Z. et al., 2019
miR-24	Human, mouse	S1PR1, H2A.X, HO-1	Promote infiltration of inflammatory cells	Lorenzen et al., 2014
miR-155	Rat	FoxO3a	Induce pro-inflammatory cytokines	Wu et al., 2016
miR-214	Mouse	mt-Nd6, mt-Nd4l	Disrupt mitochondrial oxidative phosphorylation	Bai et al., 2019
miR-351-5p	Rat, mouse	MAPK13, SIRT6	Promote oxidative stress and inflammation	Hu et al., 2018
miR-374b-5p	Mouse	Socs1	Promote M1 macrophage activation and inflammation	Ding et al., 2020
miR-494	Human, mouse	HtrA3, ATF3	Enhance renal inflammation	Lan et al., 2012; Gong et al., 2021
miR-1897-3p	Mouse	Nucks1	Modulate inflammation and renal injury	Bellinger et al., 2014
**Down-regulated by H/I**				
miR-27a	Rat	TLR4	Inhibit inflammation in renal IRI	Wang et al., 2019
miR-194	Human	Rheb	Suppress oxidative stress and inflammation	Shen et al., 2018
miR-195-5p	Rat	VEGFA	Inhibit inflammatory cytokines	Xu et al., 2020a
miR-449b-5p	Rat	HMGB1, MMP2	Reduce renal inflammation and apoptosis	Xu et al., 2020b

**TABLE 2:** H/I-induced changes of miRNAs in the heart with defined target genes.

H/I regulated miRNAs	Species	Target genes	Functions	References
**Up-regulated by H/I**				
miR-22	Rat	Sirt1, PGC1α	Promote mitochondrial oxidative damage	Du et al., 2016
miR-23a	Rat	CX43	Enhance mitophagy and myocardial I/R injury	Wang L. et al., 2021
miR-30c-5p	Rat	Bach1, SIRT1	Regulate cardiac inflammation and NF-κB signaling	Chen et al., 2020a; Sun et al., 2021
miR-181c-5p	Rat	PTPN4	Exacerbate myocardial I/R injury and NF-κB-mediated inflammation	Wang S. et al., 2020
miR-184	Rat	FBXO28	Promote myocardial inflammation and oxidative stress	Zou et al., 2020
miR-199a-214	Mouse	PPARδ	Impair mitochondrial fatty acid oxidation	el Azzouzi et al., 2013
miR-327	Rat	RP105	Enhance inflammation and NF-κB signaling	Yang et al., 2018
miR-346	Rat	NFIB	Promote myocardial inflammation and apoptosis	Yang et al., 2020
miR-361	Mouse	PHB1	Inhibit mitochondrial fission and apoptosis	Wang K. et al., 2015
miR-665	Rat	GLP1R	Promote inflammatory response and impair mitochondrial respiratory chain enzyme activity	Lin et al., 2019
**Down-regulated by H/I**				
miR-30e	Rat	SOX9	Inhibit myocardial inflammation	Cheng et al., 2021
miR-130a-5p	Mouse	HMGB2	Inhibit inflammatory injury and NF-κB signaling	Li Y. et al., 2021
miR-138	Human	PDK1	Promote mitochondrial respiration and inhibit glycolysis	Zhu et al., 2017
miR-142-3p	Porcine	IRAK-1	Attenuate myocardiac inflammatory response	Su et al., 2019
miR-147	Rat	HIPK2	Inhibit myocardial inflammation and apoptosis	Wu and Huang, 2020
miR-200a	Human, mouse	Keap1, β-catenin	Reduce inflammation, ROS production and apoptosis	Sun et al., 2016; Ma Y. et al., 2021
miR-204	Mouse	Cotl1	Inhibit myocardial inflammation and oxidative stress	Tan et al., 2020
miR-335	Rat	MAP3K2	Inhibit myocardial inflammation and apoptosis	Wang A. et al., 2021
miR-369	Rat	TRPV3	Reduce hypoxia-induced apoptosis and inflammation	Wang J. et al., 2021
miR-409-5p	Rat	USP7	Inhibit myocardial inflammation	Xue Q. et al., 2021
miR-495	Mouse	NLRP3	Inhibit NLRP3 inflammasome signaling	Zhou et al., 2018
miR-499-5p	Rat	CnAa, CnAb	Regulate mitochondrial dynamics	Wang J. et al., 2011
miR-668-3p	Rat	SDF-1	Inhibit inflammation and oxidative stress	Gao et al., 2020
miR-708	Rat	HMGB1, ADAM17	Inhibit pro-inflammatory cytokine and NF-κB signaling	Zhang et al., 2020c; Qu et al., 2021
miR-1278	Mouse	IL-22, CXCL14	Inhibit myocardial inflammation	Liu D. et al., 2021

Opioid receptors belong to the large family of seven-transmembrane G protein-coupled receptors with three major sub-types known as MOR, KOR, and DOR (Xia, 2015). It is well demonstrated that DOR is protective against hypoxic, ischemic, and excitotoxic insults. There exists a differential distribution of DOR in different parts of the brain with a higher density in the cortex, striatum and dorsal root ganglion (Xia, 2015; Tan et al., 2016; Li X. et al., 2020; Gao et al., 2021). DOR is present not only in the nervous system but also in other organs such as the heart, lungs, liver, and gastrointestinal and reproductive tracts (Feng et al., 2012). Our recent studies as well as those of others present strong evidence of the DOR-mediated cyto-protection in different organs, including brain, kidney, heart, and liver (Zhang et al., 2000, 2002, 2006; Ma et al., 2005; Chao et al., 2007, 2008, 2009, 2012; Kang et al., 2009; Chao and Xia, 2010; Feng et al., 2011, 2012; He et al., 2013a; Luo et al., 2019). Moreover, there is accumulating evidence that DOR activation can achieve a protective role against H/I injury by modulating miRNA expression in multiple organs (Yang et al., 2012; He et al., 2013b; Zhi et al., 2016, 2017), especially in the process of neuroinflammation (Chen et al., 2020c). It is therefore possible for DOR signaling, directly or indirectly, to protect organs against H/I inflammation by targeting specific miRNA molecules.

In this review, we highlight some of the recent updates about the effects of H/I and DOR activation on miRNA expression in the current literature, along with our recent work in this field. In particular, we intend to summarize and compare differential regulation of miRNA expression in different organs and discuss potential clinical significances in terms of specific DOR treatment against H/I inflammation.

## Effects of Hypoxia/Ischemia on Micrornas in Hypoxia-Sensitive Organs

### Kidney

Although blood flow to the kidney accounts for 20% of the cardiac output, the kidney is vulnerable to H/I injury because of the renal vascular anatomy and the high energy consumption of renal tubular epithelial cells (Bracken et al., 2006). H/I-induced inflammatory processes exert a significant role in the development of nephrotic diseases, such as acute kidney injury, glomerulonephritis, and chronic allograft nephropathy (Wang Z. et al., 2020; Nangaku and Eckardt, 2007; Heyman et al., 2008; Chen et al., 2020b). Moreover, the ischemia–reperfusion process during renal transplantation has a profound influence on both short- and long-term recovery outcome of a transplanted kidney (Kosieradzki et al., 2003; Kosieradzki and Rowinski, 2008; Chen et al., 2019). Several studies have suggested that H/I condition, especially a prolonged stress, could strongly influence the miRNA expression profiles in kidneys (Godwin et al., 2010; Shapiro et al., 2011). A number of altered miRNAs have been discovered during H/I kidney injury with identified target genes, which regulate cell inflammatory phenotypes (Table 1). Recent studies in human and rodent models also suggested a crosstalk among different renal cell types after H/I insult. For example, H/I-induced upregulation of miR-21 and miR-374b-5p could promote renal inflammation by activating M1 macrophage (Ding et al., 2020; Pushpakumar et al., 2021). Renal tubular epithelial cells are usually the initial site of renal injury, with activation of fibroblasts or macrophage occurring later by exosome delivery of specific miRNAs (Tan et al., 2016; Li Z. et al., 2019). Pro-inflammatory cytokine production and tubulointerstitial inflammation induced by miRNAs could eventually cause renal fibrosis (Table 1).

### Heart

Heart is a H/I-sensitive organ with active metabolism. H/I-induced myocardial injury is closely associated with cardiac disorders such as myocardial infarction and heart failure (Dirksen et al., 2007; Cassavaugh and Lounsbury, 2011; Frohlich et al., 2013). MicroRNAs have been demonstrated to orchestrate many aspects in the development of heart diseases. Recent research on miRNAs regarding myocardial inflammation has drawn much attention from clinicians and scientists. Under H/I condition, the mismatch between energy production and consumption has profound impacts on mitochondrial function and energy metabolism processes of cardiomyocytes (Table 2). Pro-inflammatory miRNAs such as miR-22, miR-199a-214, miR-361, and miR-665 are elevated (el Azzouzi et al., 2013; Wang K. et al., 2015; Du et al., 2016; Lin et al., 2019), whereas anti-inflammatory miRNAs such as miR-138 and miR-499-5p are reduced (Wang J. et al., 2011; Zhu et al., 2017) to impair normal mitochondrial function in myocardial cells. In addition, abnormal expression of miR-30c-5p, miR-181c-5p, miR-327, miR-130a-5p, and miR-708 upon H/I stimulation could regulate myocardial inflammation through NF-κB signaling (Yang et al., 2018; Chen et al., 2020a; Wang S. et al., 2020; Zhang et al., 2020c; Li Y. et al., 2021; Qu et al., 2021; Sun et al., 2021; Table 2). One of the key inflammatory mediators, NLRP3 inflammasome, may also be a direct target of H/I-sensitive miRNAs. A recent study in rodents identified that miR-495 ameliorated cardiac microvascular endothelial cell injury and inflammatory reaction by suppressing the NLRP3 inflammasome signaling pathway (Zhou et al., 2018).

### Brain

Hypoxic and/or ischemic injuries are well-documented entities in the pathogenesis of cerebrovascular diseases such as stroke. The effects of H/I on miRNAs in the brain have been widely investigated in patients as well as animal ischemic models with middle cerebral artery occlusion (MCAO). Our previous reviews have summarized the regulation of brain miRNAs in response to hypoxic and ischemic conditions (Yang et al., 2015) and their impact on neuroinflammatory signaling pathways (Chen et al., 2020c). Many studies have elucidated the possible mechanisms and the potential biological processes of H/I-induced miRNA expression changes. The neuroinflammation processes including NLRP3 signaling, mitochondrial impairment, microglia activation, inflammatory cytokines production, and potentially neurodegeneration (Chen et al., 2017, 2020c). In general, up-regulation of pro-inflammatory miRNAs and down-regulation of anti-inflammatory miRNAs are often observed in H/I brains (Table 3). The majority of dysregulated miRNAs in the brain exposed to H/I insult displayed a reduction of miRNA expression, especially after a long-term exposure. Similarly with other H/I-sensitive organs, cerebral miRNAs can directly influence inflammatory cytokines production by modulating target genes. They can also affect NLRP3 inflammasome and NF-κB signaling to modulate neuroinflammation. Moreover, the abnormal expression of cerebral miRNAs, e.g., miR-186-5p, miR-200b, miR-210, miR-449c-5p, miR-302a-3p, miR-424, miR-665-3p, and let-7c-5p, can alter the activation of microglia, the most important inflammatory cell type in the brain, and then indirectly influence neuroinflammation, neuronal death and neurodegeneration (Table 3).

**TABLE 3:** H/I-induced changes of miRNAs in the brain with defined target genes.

H/I regulated miRNAs	Species	Target genes	Functions	References
**Up-regulated by H/I**				
miR-7-5p	Rat	Sirtuin 1	Enhance cerebral inflammation	Zhao and Wang, 2020
miR-19a-3p	Human, rat	IGFBP3	Promote inflammation	Chai et al., 2020
miR-20b	Rat	NLRP3	Promote inflammation by activate NLRP3 signaling	Zhao et al., 2019
miR-21-3p	Rat	MAT2B	Promote inflammation	Li C. et al., 2019
miR-155	Human, mouse	MafB, DUSP14	Induce inflammatory mediators expression	Shi Y. et al., 2020; Zhang et al., 2020a
miR-186-5p	Rat	CTRP3	Increase microglia/macrophage inflammation	Chen et al., 2021
miR-200b	Rat	KLF4	Induce microglia M1 polarization	Wen et al., 2018
miR-210	Mouse	TET2	Induce macrophage infiltration, microglial activation and inflammation	Huang et al., 2018; Ma Q. et al., 2021
miR-217	Rat	SIRT1, MEF2D	Induce neuronal injury and inflammatory response	Rao et al., 2019; Shi L. et al., 2020
miR-449c-5p	Rat	STAT6	Promote microglial inflammation	Zhang et al., 2020b
miR-3473b	Mouse	SOCS3	Promote neuroinflammation	Wang X. et al., 2018
**Down-regulated by H/I**				
miR-7a-5p	Rat	SNCA	Inhibit mitochondrial fragmentation and oxidative stress	Kim et al., 2018
miR-17-5p	Rat	TXNIP	Inhibit NLRP3 inflammasome	Chen et al., 2018
miR-26b-5p	Rat	Smad1	Inhibit apoptosis and inflammatory responses	Shangguan et al., 2020
miR-29a	Rat	TP53INP1	Inhibit NLRP3 inflammasome	Liu X. et al., 2021
miR-34c-5p	Rat	NCOA1	Inhibit inflammatory cytokines and NF-κB signaling	Tu and Hu, 2021
miR-124	Rat	CYBB	Inhibit neuroinflammation and NF-κB signaling	Wu et al., 2020
miR-125b	Rat	TP53INP1	Inhibit neuroinflammation and apoptosis	Li et al., 2018
miR-140-3p	Rat	HIF-1α	Alleviate inflammation, oxidative stress and apoptosis	Yi et al., 2020
miR-181c-3p	Rat	CXCL1	Inhibit inflammation in astrocytes	Song et al., 2019
miR-182-5p	Rat, mouse	TLR4	Inhibit inflammatory cytokines	Wang J. et al., 2018
miR-199b	Mouse	AQP4	Inhibit neuroinflammation	Zhang et al., 2021
miR-302a-3p	Mouse	STAT1	Inhibit microglial inflammation	Hu et al., 2021
miR-367-3p	Mouse	Gprc5a	Inhibit neuroinflammation	Tabet et al., 2020
miR-374a-5p	Rat	Smad6	Inhibit pro-inflammatory cytokines and NLRP3 inflammasome	Chen et al., 2020e
miR-381	Rat	IRF4	Inhibit inflammatory cytokines	Fang et al., 2021
miR-410	Human	PTEN	Inhibit neuroinflammation	Meng et al., 2021
miR-421-3p	Mouse	YTHDF1	Prevent inflammatory response	Zheng et al., 2020
miR-424	Human, mouse	CDC25A, CCND1, CDK6	Inhibit neuronal apoptosis and microglia activation	Zhao et al., 2013
miR-485	Rat	AIM2	Inhibit pyroptosis and inflammation	Liang et al., 2020
miR-532-5p	Rat	CXCL1	Inhibit neuroinflammation and NF-κB signaling	Shi et al., 2021
miR-542-3p	Mouse	TLR4	Inhibit neuroinflammation	Cai et al., 2021
miR-665-3p	Mouse	TRIM8	Inhibit apoptosis and microglial inflammation	Zhang et al., 2020d
miR-874-3p	Human, mouse	CXCL12	Promote angiogenesis and inhibit inflammation	Xie et al., 2020
miR-1202	Human	Rab1a	Inactivate TLR4/NF-κB-involved inflammatory signaling pathway	Song et al., 2020
let-7c-5p	Human, mouse	Caspase-3	Inhibit microglia activation	Ni et al., 2015
let7i	Human	CD86, CXCL8, HMGB1	Regulate leukocyte activation, recruitment and proliferation	Jickling et al., 2016

## Effects of Hypoxia/Ischemia on Micrornas in Hypoxia-Insensitive Organs

### Liver

Although liver is regarded as one of the hypoxia-insensitive organs, oxygen is also important for liver function maintenance. The insufficiency or deprivation of oxygen and blood flow in hepatic microenvironment due to respiratory/circulatory disorders leads to hepatocellular damage. Indeed, hypoxia activates multiple hypoxia mediators and in turn accelerate or antagonize hepatic damage (Klune and Tsung, 2010; Lu et al., 2016), such as fatty liver disease (Suzuki et al., 2014), hepatocellular carcinoma (HCC) (Liu et al., 2015), and liver-stage malaria (Ng et al., 2014).

Multiple studies have illustrated the H/I injury-induced miRNA changes in liver inflammation, hepatocellular oxidative stress and apoptosis. Meanwhile, altered miRNAs are closely associated with hypoxia-induced HCC pathogenesis by modulating HCC cell angiogenesis, viability and metastasis (Table 4). For example, the hypoxia-sensitive miR-210, which is regarded as the master orchestrator miRNA to H/I insults, is significantly up-regulated in the liver. It mediates hypoxia-induced liver inflammation and HCC cell metastasis through targeting VMP1 (Ying et al., 2011; Song et al., 2014). In addition, hypoxia elevated miR-370 expression and thus promoted inflammation and hepatic histological damage by targeting TGFBR2 (Li et al., 2015). In contrast, cyto-protective miRNAs including miR-24-3p, miR-140-5p, miR-142-3p, miR-146a, and miR-148a were down-regulated under H/I condition, and thus impaired liver function by elevating pro-inflammatory responses (Table 4).

**TABLE 4:** H/I-induced changes in miRNAs in H/I-insensitive organs with defined target genes.

Organ	H/I regulated miRNAs	Species	Target genes	Functions	References
**Liver**	**Up-regulated by H/I**				
	miR-210	Human	VMP1	Mediate hypoxia-induced HCC cell metastasis and liver inflammation	Ying et al., 2011; Song et al., 2014
	miR-370	Mouse	TGFBR2	Induce proinflammatory cytokines and hepatic histological damage	Li et al., 2015
	miR-450b-5p	Mouse	CRYAB	Induce inflammatory cytokines and inhibit macrophage M2 polarization	Huang et al., 2020
**Down-regulated by H/I**					
	miR-24-3p	Mouse	STING	Inhibit inflammatory response and apoptosis in hepatic I/R process	Shen et al., 2020
	miR-128-3p	Mouse	Rnd3	Activate NF-κB signaling	Mou et al., 2020
	miR-140-5p	Mouse	CAPN1	Inhibit inflammatory response and apoptosis	Yu et al., 2021
	miR-142-3p	Mouse	MARCKS	Attenuate hepatic I/R injury and inflammation	Li Y. et al., 2020
	miR-146a	Mouse	IRAK1, TRAF6, TLR4	Inhibit proinflammatory cytokines release and apoptosis	Jiang et al., 2014; Chungen et al., 2020
	miR-148a	Mouse	CaMKIIα	Inhibit TLR4-mediated inflammation	Zheng et al., 2018
**Muscle**	**Up-regulated by H/I**				
	miR-93	Mouse	IRF9	Induce M2-like macrophage polarization in ischemic muscle	Hazarika et al., 2013; Ganta et al., 2017
	miR-155	Human	SOCS-1	Aggravate inflammatory response, leukocyte infiltration and tissue damage	Eisenhardt et al., 2015
**Down-regulated by H/I**					
	miR-92b-3p	Rat	HIF1A	Inhibit inflammatory cytokines	Wang and Quan, 2021
	miR-98	Human	IL6	Modulate inflammation and PASMC apoptosis	Wang Q. et al., 2015
	miR-146b	Mouse	TRAF6	Inhibit inflammatory factors expression	Desjarlais et al., 2019
	miR-let-7a	Human	STAT3	Inhibit inflammation	Cheng et al., 2020

*PASMC, pulmonary artery smooth muscle cells.*

### Muscle

In comparison to other organs, muscles are relatively tolerant to hypoxic and ischemic stress. Like other H/I-sensitive organs, however, H/I-stimulated muscles also regulate several molecular pathways to better adapt to hypoxic/ischemic environments (Zhu et al., 2022). Recent studies have been devoted to underpinning the mechanisms and pathways of miRNA alternations upon ischemia–reperfusion injury, including macrophage polarization, leukocyte infiltration, and pro-inflammatory cytokine production in ischemic muscle (Table 4). It is reported that miR-93 inhibits IRF9 and induces M2-like polarization in ischemic muscles to enhance angiogenesis, arteriogenesis, and perfusion recovery in peripheral artery disease (Ganta et al., 2017). In *in vitro* studies, pulmonary artery smooth muscle cells (PASMCs) have been used to investigate H/I-induced muscle miRNA alternations. An H/I down-regulation of miR-98 was observed to modulate inflammation and PASMC apoptosis by directly targeting pro-inflammatory cytokine IL6 (Wang Q. et al., 2015).

The information on H/I muscle miRNAs are still limited at present. Altered miRNAs could significantly affect muscle function and change the status of some muscle diseases by targeting key components in the inflammatory pathways. Therefore, it is needed to further investigate the regulation of muscle miRNAs in H/I condition.

## Effects of δ-Opioid Receptor Activation on Microrna Expression Profiles in Different Organs Under Normoxia

δ-Opioid receptor is neuroprotective against H/I injury in the brain (Zhang et al., 2002; Chao et al., 2008, 2009; Kang et al., 2009; He et al., 2013a). The administration of DOR agonists can prolong survival of peripheral organs, such as lung, heart, liver, and kidney (Peart et al., 2005; Patel et al., 2006). As a protective molecule, DOR can tonically regulate miRNA expression even in the normoxic condition. We measured miRNA expression profiles in different organs such as the kidney, heart, brain, and liver after DOR activation with a specific and potent DOR agonist UFP-512 applied in Sprague Dawley rats (Yang et al., 2012; He et al., 2013b; Zhi et al., 2016, 2017). DOR activation can influence the expression of many miRNAs in different organs. As summarized in Table 5, the brain had the most dramatic changes in miRNAs after DOR activation. Conversely, miRNAs in the heart and liver kept relatively stable during the observation (DOR activation for 1, 5, and 10 days), suggesting a differential regulation of miRNAs among organs in response to DOR signals under normoxic condition.

**TABLE 5:** DOR-activation induced changes in miRNA expression profiles in normoxic condition.

Organs	Kidney	Brain	Heart	Liver
**miRNAs**	let-7f	miR-21	miR-107-3p	miR-107-3p
	miR-20b-5p	miR-29a	miR-128-3p	miR-122-5p
	miR-21	miR-29b	miR-141-3p	miR-146a-5p
	miR-29b	miR-31	miR-350	miR-182
	miR-212	miR-101b		miR-184
	miR-298	miR-186		miR-192-5p
	miR-347	miR-298		
	miR-351	miR-324-3p		
	miR-370	miR-347		
	miR-466b	miR-351		
	miR-511	miR-363*		
		miR-466b		
		miR-511		

*Summarized from our published articles (Yang et al., 2012; He et al., 2013b; Zhi et al., 2016, 2017).*

Among hypoxia-sensitive organs, seven common miRNA changes were observed in the kidney and brain, including miR-21, miR-29b, miR-298, miR-347, miR-351, miR-466b, and miR-511 (Table 5; Yang et al., 2012; He et al., 2013b). MicroRNAs in the kidney displayed the similar expression tendency with those of the brain. For instance, miR-29b was significantly down-regulated in the brain and kidney at 1 day after DOR activation with UFP-512 and maintained at a relatively stable level after 5–10 days DOR activation. Reduced expression level of miR-347 was observed after DOR activation at day 1 in the brain, and at days 5 and 10 in the kidney. Similarly, miR-511 was significantly down-regulated after DOR activation in rat brain and kidney at day 10 (Tables 6, 7). In contrast, there were few common miRNA changes among hypoxia-insensitive organ (liver) and hypoxia-sensitive organs. In our work, only the alternation of miR-107-3p expression was seen in both the heart and liver in normoxic condition upon DOR activation (Table 5). Further investigating their functions and elucidating the mechanistic differences between the H/I sensitive and -insensitive organs will yield valuable information for better understand the DOR-mediated regulation of miRNAs in physiological conditions.

**TABLE 6:** Effects of DOR activation on brain miRNAs in prolonged hypoxia.

miRNA	1 day			5 days			10 days		
	Hypoxia	C + DOR	H + DOR	Hypoxia	C + DOR	H + DOR	Hypoxia	C + DOR	H + DOR
miR-29b	↓	↓	–	↓	–	–	–	–	–
miR-324-3p	↓	↓	–	↓	–	–	–	–	–
miR-347	↓	↓	↓	↓	–	↓	–	–	↓
miR-298	↓	↓	–	↓	–	↓	–	–	–
miR-101b	↓	↓	↓	–	–	↓	–	–	–
miR-466b	↓	↓	↓	↓	–	↓	–	–	–
miR-186	–	–	–	↓	↓	↓	–	–	–
miR-20b-5p				–	–	↓			
miR-212				–	–	↓			
miR-351				–	↑	↓			
let-7f							↓	–	–
miR-29a	–	↓	↓				↓	–	–
miR-511				–	↓	–	↓	↓	–
miR-363*	–	↑	↓	–	–	↓	–	↓	–
miR-370	–	–	–	↓	–	↓	–	–	–
miR-21	–	↓	–	–	–	↓	–	↑	↓
miR-31	–	–	↑	↓	–	↓	–	↓	–

*↑, up-regulation; ↓, down-regulation; –, no statistical difference; C, normoxic control; H, hypoxia; DOR, DOR activation. Comparisons: hypoxia vs. C; C + DOR vs. C; H + DOR vs. H. Summarized from our published article (Yang et al., 2012).*

**TABLE 7:** Effects of DOR activation on renal miRNAs in prolonged hypoxia.

miRNA	1 day			5 days			10 days		
	Hypoxia	C + DOR	H + DOR	Hypoxia	C + DOR	H + DOR	Hypoxia	C + DOR	H + DOR
let-7f							↓	↓	–
miR-363*							↓	–	↑
miR-370							–	↑	↓
miR-466b							↓	↓	↑
miR-511							↓	↓	↓
miR-298				–	↑	↓	↑	–	↓
miR-324-3p				↑	–	–	↑	–	↓
miR-20b-5p				–	–	↓	–	↑	–
miR-347	–	–	–	–	↓	↓	↓	↓	–
miR-212	–	–	–	–	↓	↓	↓	↓	–
miR-351	–	↑	–	–	↓	–	↓	–	–
miR-29a	–	–	↓	–	–	↓	↑	–	–
miR-21	↓	↓	↓	–	–	↓	–	↓	–
miR-29b	↓	↓	↓	–	–	↓	–	–	↓

*↑, up-regulation; ↓, Down-regulation; -, No statistical difference; C, normoxic control; H, hypoxia; DOR, DOR activation. Comparisons: hypoxia vs. C; C + DOR vs. C; H + DOR vs. H. Summarized from our published article (He et al., 2013b).*

## Effects of δ-Opioid Receptor Activation on Microrna Expression Profiles in Different Organs Under Hypoxia

Since either DOR activation or hypoxic condition has a profound impact on miRNA expression profiles in different organs, it will be interesting to learn the possible effect of DOR activation on hypoxia-induced miRNA changes. Our studies have shown that miRNA expression profiles can be significantly altered when DOR activation was applied to hypoxic organs (Yang et al., 2012; He et al., 2013b; Zhi et al., 2016, 2017). We noticed that the differential alternations in the miRNAs largely depended on the duration of hypoxia, and DOR activation led to diverse outcomes in response to short-term or prolonged hypoxia in different organs.

As one of the hypoxia-sensitive organs, the hypoxic brain showed the most dramatic changes in miRNA expression in response to DOR activation. Some miRNAs were largely altered at the earliest time point (1 day), including miR-347, miR-101b, miR-466b, miR-29a, miR-363*, and miR-31 (Yang et al., 2012). The majority of miRNAs, especially those that mediates cyto-protective function such as anti-neuroinflammation, were down-regulated compared with those in normal condition. Among them, miR-29a alleviated cerebral ischemia/reperfusion injury via down-regulating target gene TP53INP1 and the NF-κB/NLRP3 pathway (Liu X. et al., 2021). In the brain exposed to a mid-term hypoxia (5 days), DOR activation provoked even more miRNA reduction as compared to that in short-term hypoxia (1 day), whereas the expression of miRNAs turned to the baseline level upon DOR activation under prolonged hypoxia (Table 6; Yang et al., 2012). These findings implicated that brain miRNA expression responded quickly after the hypoxia insult, and possibly involved in anti-hypoxic injury by modulating the expression of target genes.

δ-Opioid receptor activation can also modify hypoxia-induced changes of miRNA expression in the kidney. The changes in several miRNAs could be detected upon DOR activation in the kidney exposed to a continuous hypoxia for a prolonged period. For instance, the down-regulation of miR-511 and miR-298 expression was observed upon DOR activation followed by a continuous exposure to hypoxia for 5 to 10 days (He et al., 2013b). In the case of miR-370, miR-20b-5p, and miR-29a/b, although hypoxia alone did not alter their expression levels at certain time points, the administration of UFP-512 to the hypoxic kidney significantly down-regulated the expression of these miRNAs. MiR-21 was reported to induce macrophage M1 inflammatory phenotype, cytokine production, endothelial-mesenchymal transition, and fibrosis in ischemic kidney (Pushpakumar et al., 2021). DOR activation could lower miR-21 expression in prolonged hypoxia (5 days and 10 days). Apparently, DOR activation continuously modulated renal miRNA expression during the whole hypoxic period (Table 7; He et al., 2013b).

Unlike other hypoxia-sensitive organs, the majority of altered miRNAs in the heart was up-regulated under 1 day hypoxia. The administration of UFP-512 to the hypoxic heart induced a further upregulation in terms of the expression of miR-141-3p, miR-376a-3p and miR-134-5p. Moreover, the expression of miR-134-5p and miR-7b was increased throughout the entire time course after DOR activation under hypoxia, suggesting their important roles in the regulation of renal adaptation to hypoxic stress (Table 8; Zhi et al., 2016).

**TABLE 8:** Effects of DOR activation on cardiac miRNAs in prolonged hypoxia.

miRNA	1 day			5 days			10 days		
	Hypoxia	C + DOR	H + DOR	Hypoxia	C + DOR	H + DOR	Hypoxia	C + DOR	H + DOR
miR-7a-5p	↑			–			↓		
miR-141-3p	↑	↑	↑	–	–	–	–	–	–
miR-196c-5p	↑			–			↓		
miR-200a-3p	↑			↑			–		
miR-200b-3p	–		↑	–		–	–		–
miR-203a-3p	↑			–			–		
miR-324-3p	↑			–			↓		
miR-376a-3p	↑		↑	–		↑	–		–
miR-135a-5p	↑			↑			↑		
miR-193a-3p	↑			↑			–		
miR-338-3p	↑			↑			–		
miR-128-3p	–	–		–	–		–	↑	
miR-134-5p	–		↑	↑		↑	↑		↑
miR-350	–	↑		–	–		↓	–	
miR-107-3p		↑	↑		↑	–		–	–
miR-7b	–		↑	–		↑	–		↑

*↑, up-regulation; ↓, down-regulation; –, no statistical difference; C, normoxic control; H, hypoxia; DOR, DOR activation. Comparisons: hypoxia vs. C; C + DOR vs. C; H + DOR vs. H. Summarized from our published article (Zhi et al., 2016).*

Similar to that in hypoxia-sensitive organs, miRNA expression in hypoxia-insensitive organs can also be influenced by DOR activation in hypoxic condition. The expression of miR-34a-5p, miR-142-5p, miR-145-5p, miR-146a-5p, and miR-204-5p were significantly increased in the liver after DOR activation and hypoxic stress (Zhi et al., 2017). MiR-192-5p was the only miRNA whose expression level started to decrease after DOR activation under hypoxia (Table 9). MiR-146a was reported to ameliorate ischemia/reperfusion injury *in vivo* and hypoxia/reoxygenation injury *in vitro* by directly suppressing IRAK1 and TRAF6 in the liver (Jiang et al., 2014). Prolonged hypoxia could down-regulate miR-146a level, whereas DOR activation restored miR-146a expression, which might inhibit pro-inflammatory cytokine release and cellular apoptosis. Therefore, DOR signaling likely functions to upregulate hepatic tolerance to hypoxic stress by differentially modulating the expression of different miRNAs.

**TABLE 9:** Effects of DOR activation on liver miRNAs in prolonged hypoxia.

miRNA	1 day			5 days			10 days		
	Hypoxia	C + DOR	H + DOR	Hypoxia	C + DOR	H + DOR	Hypoxia	C + DOR	H + DOR
miR-7a-5p	↑			–			–		
miR-10a-5p	↑			↑			↓		
miR-25-3p	↑			↑			–		
miR-26b-5p	↑			↑			–		
miR-30e-5p	↑	–		↓	–		–	–	
miR-34a-5p	↓		↑	↓		↑	↓		↑
miR-34c-5p	↑			↑			↑		
miR-107-3p	↑	–		↑	↓		↑	↓	
miR-122-5p	↑	↓		↑	↓		–	↓	
miR-128a-3p	↑	–		↑	–		↑	–	
miR-135b-5p	↑			–			–		
miR-142-5p	↑		↑	↓		↑	↓		↑
miR-145-5p	↑		↑	↑		–	–		↑
miR-146a-5p	–	–	↑	↓	↑	↑	↓	↑	↑
miR-181a-5p	↑			–			↓		
miR-182	↓	↑		↓	↑		↓	–	
miR-184	–	–		–	↓		–	↓	
miR-192-5p	↑	↑	↓	↑	↑	↓	↑	↑	↓
miR-204-5p	↑		↑	–		↑	–		↑

*↑, up-regulation; ↓, down-regulation; –, no statistical difference; C, normoxic control; H, hypoxia; DOR, DOR activation. Comparisons: hypoxia vs. C; C + DOR vs. C; H + DOR vs. H. Cited from our previous work (Zhi et al., 2017).*

Collectively, among hypoxia-sensitive organs, the kidney and brain had a common change in the miRNAs, i.e., a significant alternation in nine miRNAs in both organs (Yang et al., 2012; He et al., 2013b), whereas hypoxia-sensitive and -insensitive organs had no common change at all (Table 10).

**TABLE 10:** DOR-activation modifies hypoxia-induced changes in miRNA expression.

Organs	Kidney	Brain	Heart	Liver
**miRNAs**	miR-20b-5p	miR-20b-5p	miR-7b	miR-34a-5p
	miR-21	miR-21	miR-107-3p	miR-142-5p
	miR-29a	miR-29a	miR-134-5p	miR-145-5p
	miR-29b	miR-31	miR-141-3p	miR-146a-5p
	miR-212	miR-101b	miR-200b-3p	miR-192-5p
	miR-298	miR-186	miR-376a-3p	miR-204-5p
	miR-324-3p	miR-212		
	miR-347	miR-298		
	miR-363*	miR-347		
	miR-370	miR-351		
	miR-466b	miR-363*		
	miR-511	miR-370		
		miR-466b		

*Summarized from our published articles (Yang et al., 2012; He et al., 2013b; Zhi et al., 2016, 2017).*

It seems that hypoxia comprehensively modifies miRNA profiles with a major difference among organs, while DOR signaling is able to modulate such regulation in most of these organs (Feng et al., 2012; He et al., 2013a; Nallamshetty et al., 2013; Yang et al., 2015). DOR activation may induce cyto-protection against H/I insult in both hypoxia-sensitive and -insensitive organs, at least partially, through modulating miRNA expression.

## Conclusion and Pharmacological Perspectives

Hypoxic/ischemic-induced inflammatory injury to different organs is a frequently encountered clinical problem and the common cause of various diseases with limited therapeutic options. Because the role of miRNAs in controlling H/I inflammation, recent studies on miRNA expression under H/I condition have drawn much attention from clinicians and scientists worldwide. Many experiments have been conducted in cell models, animal models, and patients to investigate the potential targets and signaling pathways of miRNAs involved in H/I pathology. Some of the miRNAs are regarded as injury factors under hypoxic condition by promoting cellular inflammation, mitochondrial dysfunction, oxidative stress and apoptosis, while others play protective roles against H/I insult by inhibiting pro-inflammatory cytokines release and NLRP3 inflammasome (Tables 1–4). Our summary of increased or decreased miRNAs in response to DOR activation (Tables 5, 10) provides a guide for future clarifications of their functions in controlling H/I inflammation.

Hypoxic/ischemic stress comprehensively alters miRNA expression in H/I-sensitive and -insensitive organs, largely depending on the duration of hypoxia. MicroRNA expression in H/I-sensitive organs such as brain and kidney often respond quickly to relatively short-term hypoxia (1 day). After prolonged hypoxia (10 days), the expression of some miRNAs turned back to the baseline level. There is evidence suggesting that a mild/moderate H/I stress may induce pro-inflammatory cytokine release and causes a quick inflammatory response, whereas a severe/prolonged stress eventually causes cell apoptosis and necrosis (Krock et al., 2011; Hadjipanayi and Schilling, 2013). The differential cellular signaling is partially mediated by the miRNA-induced repression of gene expression.

Although various miRNAs can exhibit very different responses even in the same organ under similar H/I condition, there are several H/I sensitive miRNAs that display common changes among different organs. These miRNAs were named “hypoxamiRs,” including miR-21, miR-210, and miR-199a (Nallamshetty et al., 2013; Greco et al., 2014; Azzouzi et al., 2015; Bertero et al., 2017). Accumulating evidence shows that the expression of miR-21 can be directly induced by hypoxia stimulation in several cell types due to the consensus hypoxia-response-element (HRE) sequence in its promoter region (Kulshreshtha et al., 2007; Parikh et al., 2012). Similarly, the expression of miR-210 could be up-regulated by either HIF-dependent mechanism via HRE binding (Lee et al., 2009; Pocock, 2011), or HIF-independent transcriptional regulations (Bertero et al., 2017). One of the most important functions mediated by these “hypoxamiRs” is cellular inflammation (Tables 1–4).

Aging is a key factor affecting H/I inflammation and miRNA expression. In contrast to the immature and young organs, the aged ones are more sensitive to H/I stress. The oxygen-deprived conditions (hypoxia and ischemia) lead to oxidative stress, cellular damage and protein modifications (Adav and Sze, 2020). H/I inflammation and abnormal miRNA expression have been proposed as risk factors for aging and neurodegenerative diseases. Some circulating inflamma-miRs, e.g., miR-21-5p and miR-126-3p, are even thought as potential biomarkers of cognitive impairment AD patients (Giuliani et al., 2021). MicroRNAs implicated in pathological aging such as miR-92a-5p and miR-532-5p are also regarded as potential biomarkers and putative molecular effectors of cognitive frailty (Carini et al., 2021). Serum miR-214 as well as salivary miR-874 and miR-145-3p might serve as auxiliary biomarkers for PD (Chen et al., 2020d; Li L. et al., 2021). In aged rats, transcription factor HIF could protect against ischemic brain injury by reducing inflammatory responses via the Akt signaling pathway (Du et al., 2020). All these facts suggest that an elevated level of inflammation exists in various aging-related chronic diseases, and modulating miRNA expression is a promising avenue for the prevention and treatment of aging and chronic diseases.

The miRNA expression can be modulated by sequence-specific miRNA mimics (or agomirs) and inhibitors (or antagomirs), both *in vitro* and *in vivo*. A recent study showed that miR-363-3p treatment attenuates brain ischemia-induced long-term cognitive deficits in rats (Panta et al., 2020), suggesting the potential applications of miRNA mimics. The process of miRNA biogenesis involves transcription, pre-miRNA splicing, exporting, and stability of mature miRNAs. Therefore, the miRNA expression can be modulated by specific molecular signals.

The information in this review suggests that DOR activation is effective in protecting organs against H/I injury, with the capability of modulating miRNAome in both normoxic and hypoxic conditions. Although the direct regulatory mode of DOR on miRNA expression is still unknown, DOR signaling may affect miRNA biogenesis by modulating some key transcriptional factors. For instance, ERK is found to suppress pre-miRNA export from the nucleus to cytoplasm through phosphorylation of Exportin-5, resulting in a global reduction of pre-miRNA loading and miRNA synthesis (Xie et al., 2020; Zhang et al., 2020d). Since ERK activity could be upregulated by DOR activation (Ma et al., 2005; Cai et al., 2021), DOR may regulate miRNA expressions via this signaling pathway.

Different kinds of DOR agonists have been developed in the past. Many of them displayed analgesic, antidepressant, anxiolytic and other opioid effects. For instance, Deltorphin I is an opioid peptide with relatively high affinity and selectivity to DOR and produce centrally mediated analgesic effects in animals (Thomas et al., 1997). Another commonly used one is delta opioid peptide DADLE ([D-Ala2, D-Leu5]-Enkephalin), which was used for pre-conditioning and post-conditioning to induce neuroprotection against hippocampal injury resulted from transient forebrain ischemia in rats (Wang S. et al., 2011). There are also other non-peptide DOR agonists used for antidepressant, anxiolytic or anti-inflammatory properties, including SNC-80 (Bilsky et al., 1995; Perrine et al., 2006), AZD2327 (Hudzik et al., 2014), and ADL5859 (Nozaki et al., 2012). In addition, AR-M 1000390 (Wei et al., 2000) and DPI-3290 (Ananthan, 2006) were also used for DOR agonists. However, UFP-512 that we commonly used in our studies might yield more reliable data for DOR activation because of its specificity and potent binding affinity (Balboni et al., 2002; He et al., 2013b; Xia, 2015). Future development of more specific DOR agonists with lower side-effects may facilitate the application of DOR for the treatment of H/I inflammation.

In summary, it is possible to develop a new protective strategy against H/I injury by activating DOR signaling and targeting certain miRNAs to suppress H/I inflammation in both hypoxia/ischemia-sensitive and -insensitive organs. However, controversies and ambiguity still persist in the literature, especially regarding the up-regulation versus down-regulation of miRNAs under different conditions and their downstream targets in the different organs. Moreover, the molecular mechanisms involved in the DOR-mediated regulation of miRNAs are largely unknown at present. The controversies may be partially attributed to the differences in the models, species, and experimental approaches among different studies. Nevertheless, the solution of the above-mentioned fundamental issues depends on more reliable and in-depth studies in future.

## Author Contributions

YX initiated the project and made the outline. YC searched the literature and prepare the manuscript. YH, SZ, and XH participated in writing the manuscript. YX and DX supervised the project and revised the manuscript. All authors contributed to the article and approved the submitted version.

## Conflict of Interest

The authors declare that the research was conducted in the absence of any commercial or financial relationships that could be construed as a potential conflict of interest.

## Publisher’s Note

All claims expressed in this article are solely those of the authors and do not necessarily represent those of their affiliated organizations, or those of the publisher, the editors and the reviewers. Any product that may be evaluated in this article, or claim that may be made by its manufacturer, is not guaranteed or endorsed by the publisher.
